# Proposed objective scoring algorithm for walking performance, based on relevant gait metrics: the Simplified Mobility Score (SMoS™)—observational study

**DOI:** 10.1186/s13018-021-02546-8

**Published:** 2021-07-01

**Authors:** Callum Betteridge, Ralph Jasper Mobbs, Daniel Ho

**Affiliations:** 1 NeuroSpine Surgery Research Group (NSURG), Sydney, Australia; 2 NeuroSpine Clinic, Prince of Wales Private Hospital, Suite 7, Level 7, Randwick, NSW 2031 Australia; 3 Faculty of Medicine, University of New South Wales, Sydney, Australia; 4 Wearables and Gait Assessment Group (WAGAR), Sydney, Australia

**Keywords:** Walking, Gait analysis, Walking speed, Gait speed, Step count, Daily step count, Objective, Health metrics, Gait metrics

## Abstract

**Background:**

Walking is a fundamental part of living, and its importance is not limited by age or medical status. Reduced walking speed (WS), or gait velocity, is a sign of advancing age, various disease states, cognitive impairment, mental illness and early mortality. Activity levels, as defined in the literature as “daily step count” (DSC), is also a relevant measure of health status. A deterioration in our walking metrics, such as reduced WS and DSC, is associated with poor health outcomes. These objective measures are of such importance, that walking speed has been dubbed “the 6th vital sign”. We report a new objective measure that scores walking using the relevant metrics of walking speed and daily step count, into an easy-to-understand score from 0 (nil mobility) to 100 (excellent mobility), termed the Simplified Mobility Score (SMoS™). We have provided equal weighting to walking speed and daily step count, using a simple algorithm to score each metric out of 50.

**Methods:**

Gait data was collected from 182 patients presenting to a tertiary hospital spinal unit with complaints of pain and reduced mobility. Walking speed was measured from a timed walk along an unobstructed pathway. Daily step count information was obtained from patients who had enabled step count tracking on their devices. The SMoS of the sample group were compared to expected population values calculated from the literature using 2-tailed Z tests.

**Results:**

There were significantly reduced SMoS in patients who presented to the spinal unit than those expected at each age group for both genders, except for the 50–59 age bracket where no statistically significant reduction was observed. Even lower scores were present in those that went on to have surgical management. There was a significant correlation of SMoS scores with subjective disability scores such as the Oswestry Disability Index (ODI) and Visual Analogue Scale (VAS) in this cohort.

**Conclusions:**

The SMoS is a simple and effective scoring tool which is demonstrably altered in spinal patients across age and gender brackets and correlates well with subjective disability scores. The SMoS has the potential to be used as a screening tool in primary and specialised care settings.

**Supplementary Information:**

The online version contains supplementary material available at 10.1186/s13018-021-02546-8.

## Background

Walking is a fundamental part of living, and its importance is not limited by age, race or medical status [1]. It represents a complex activity requiring the interplay of the visual, musculoskeletal and neurological systems [2]. As a result, complex measures of walking quality have been created which utilise 3D motion capture systems or inertial measurement unit devices to quantify how various diseases affect one’s ability to walk. However, studies have overlooked the far simpler and more available smart devices most patients carry which provide limited data regarding day-to-day walking quality.

Numerous metrics have been described to assess gait, such as walking speed, cadence and stride/step length. While these metrics have been validated through controlled situations in gait research laboratories, there are many barriers limiting the applicability of these metrics as a marker for general health. Notably, few of these metrics can be feasibly measured outside of the controlled setting of a gait laboratory. In addition, the measurement of metrics such as walking speed, cadence and step length are confounded by the Hawthorne effect when tested in an observed setting and therefore are unlikely to represent a person’s true walking and functional status [3].

On the other hand, quantification of walking by daily step count (DSC) is still a relevant marker of an individual’s physical activity and is accessible for all patients who carry smart devices such as smartwatches, fitness trackers and smartphones. The second measure of a patient’s walking capacity is walking speed (WS), measured as metres per second (m/s) which is measured by most activity trackers, fitness watches or smartphones, giving insight into a patient’s daily walking patterns without the influence of the Hawthorne effect. Both metrics have literature backing their importance as measures of general health. Reduction in WS is a key characteristic of ageing and frailty [4], as well as a predictor of falls [5], a finding in many neurological diseases [1, 6] and a predictor of mortality regardless of age [7]. Middleton, Fritz and Lusardi [2] proposed WS as the sixth functional vital sign, with a speed of over 1.35 m/s usually being associated with complete functional independence. Conversely, increasing daily activity, measured by DSC, is linked to lower all-cause mortality by reducing the incidence of metabolic syndrome and related diseases [8]; it is itself impacted in many disease states. Typically, a DSC of 10,000 or above indicates an “active” individual who is able to engage in the necessary amount of physical activity [9]. A person’s drive to engage in physical activity is clearly affected by their musculoskeletal and neurological health. In addition, the psychological and physiological burden of other illnesses, such as cancer [10] and cardiovascular disease [8], has been demonstrated to reduce activity as measured by DSC. Serious mental illness, especially depression, is associated with slower WS and physical activity (reflected in a low DSC) compared to the general population [11]. Poor lifestyle behaviours, side effects of psychoactive medication, and the impact of mental illness on motivation are all contributing factors to a reduction in gait metrics [12]. Wearable devices can act as a supporting tool to encourage fitness in people with mental illness through self-monitoring, encouragement and lifestyle coaching [13].

An objective assessment of walking would be of significant benefit for physicians to monitor a patient’s overall health, in conjunction with other routine health metrics and vital signs. Objective outcome assessments overcome limitations of subjective; patient-reported outcome measures, which suffer from poor reliability; recall and reporting bias [14]; and a lack of capacity for continuous assessment [15]. The availability of a quick and easy tool, which is objective and collected with minimal intrusion to the patient’s life to avoid bias from the Hawthorne effect, would help clinicians to rapidly screen, using a single score, a patient’s general health status. We propose a simple objective metric that combines WS and DSC which may act as a framework for objective assessment of pre- and post-intervention outcome and recovery, following various physical medical mental health or surgical interventions. In addition, such a score may act as a tool for population and regional health assessment. The data required to calculate this metric can be collected from activity trackers [16] built into fitness watches and smartphones, with some early reports of devices collecting these metrics in day-to-day living [17, 18].

Objective monitoring and the use of wearable and smart devices for data capture of gait metrics have driven a paradigm shift from the “subjective” to the “objective” era of patient outcome analysis in the clinical setting [19]. Simple scores such as the SMoS may assist the rapid identification of individuals, or indeed populations, with declining health, facilitating early intervention, which may delay the typical increased healthcare costs and diminished quality of life associated with ageing and frailty. Widespread adoption would also allow the development of population-based health interventions to improve these metrics on a broader scale.

This study aims to introduce and test the SMoS in a sample of patients with spinal pathologies and compare them to population samples in order to validate the tool as a simple screening tool for deterioration in walking quality. Given that walking quality is diminished in a number of disease states, the experimental group is expected to have lower SMoS scores across age and gender strata than the population norms. This will introduce the SMoS as a simple measure of walking quality that is quickly and easily obtained from data captured by a patient’s smart device, thus providing additional clinical information without sacrificing time.

## Declarations

Ethics approval for the present study was obtained from the South Eastern Sydney Local Health District Human Research Ethics Committee reference number 17/184.

Verbal consent for publication was obtained from each patient prior to participation.

The dataset supporting the conclusions of this article is included within the article and its additional files.

The authors declare no competing interests regarding the materials, data and outcomes of the present study.

The authors declare that there was no external funding for the present study.

All authors contributed equally to the manuscript. RM conceptualised the study and CB and RM collected data. CB performed the data analysis.

## Methods

We propose a simple metric, potentially measurable with smart devices used by most of the population. This metric, termed the Simplified Mobility Score (SMoS™), can be measured using the daily step count and walking speed obtained from smart devices like the Apple iPhone, Apple Watch, Android devices or similar products. Both gait velocity and step count are given a score out of 50 using a linear calculation with an upper limit, calculated as a percentage of the upper limit, and multiplied by 50 (Table 1). The sum of the two scores is the overall SMoS score. The upper limit of 50 was chosen to delineate those with functional disability from those without any limitation to their daily physical and functional activities who would be expected to have no negative outcomes resulting from impacted gait.

**Table 1:** Calculation of the SMoS based on the primary gait metrics of WS and DSC

Walking speed (WS)		Daily step count (DSC)	
WS (*v*)	Points (A)	DSC	Points (B)
*v* < 1.35 m/s	(*v*/1.35) × 50	DSC < 10,000	(DSC/10,000) × 50
*v* > 1.35 m/s	50	DSC > 10,000	50
SMoS = A + B			

The present study was a retrospective observational study using a database of 450 consecutive patients (aged 30 and over) presenting for the first time to a single spinal neurosurgery clinic with pain and/or sensorimotor deficits between 2017 and 2020. Each patient was consented to the study and completed a questionnaire with demographic information and disability scores (ODI, NDI, VAS). Patients were taken for a timed walk along an unobstructed pathway over a self-selected distance (30, 60, 120 or 200 metres) to measure gait velocity. DSC was obtained from their smart device based on the data over the last month of tracking. Gait data was available for 182 patients. Patients who had undergone surgery following their initial consultation but before July 2020 were considered “surgical”. Patients were excluded if they were unable to walk independently without a device or human assistant, and if they were under the age of 18.

The sample data for walking speed and daily step count were compared to expected population values obtained from large population studies measuring walking speed (*n* = 23,111) [4] and daily step count (*n* = 717,527) [20]. Two-tailed *z*-tests were used to test for statistically significant differences between the sample data and the population values, with significance defined at *p* < 0.05. Subgroup analyses were also performed by age group and gender. A two-tailed independent sample z-test was performed to determine whether there was a statistically significant difference in the mean SMoS score between patients that underwent surgery and patients that did not. The Shapiro-Wilk test for normality was performed to maintain the assumptions of the chosen statistical test. Pearson’s correlation analysis was used to determine the association of SMoS with ODI, NDI and VAS scores. Data was collected and processed using IBM SPSS Statistics, version 26.

## Results

**Fig. 1:**
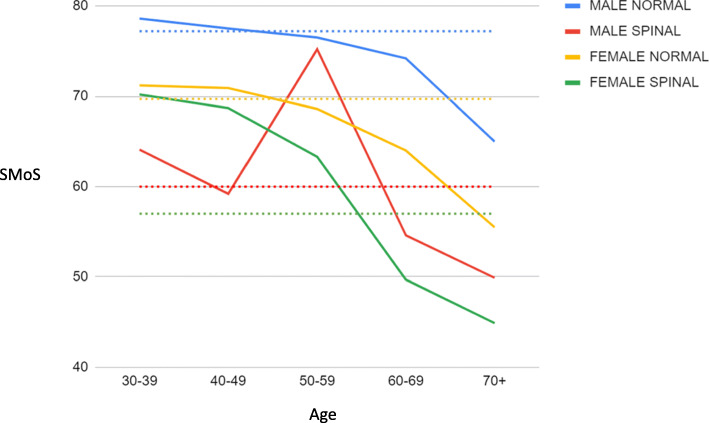
The mean SMoS scores across age, gender and pathological subgroups. Dotted lines represent the mean values across the groups

Ninety-two women and 90 men were eligible for analysis and calculation of their SMoS. The mean age was 56 years (range 20–88), and 38 (21%) had surgical intervention for their spinal pathology within the study period; the average time until surgery was 2 months. The mean ODI was 40 (range 0–98), NDI was 26 (range 0–68) and VAS was 7 (range 0–10). Figure 1 displays the population mean of SMoS within age, gender and pathological subgroups. The results of statistical analysis are displayed in Table 2.

**Table 2:** Statistical analyses for the reduction in SMoS in spine patients compared to expected population values, separated by age group and gender

Age group	Population	Sample		Difference	
	Mean (± SD)	Mean (± SE)	n	Mean difference (95% CI)	P
Total	74.3 ± 3.63	58.7 ± 1.68	182	− 15.6 (− 18.90 to − 12.30)	< .001
Male	77.2 ± 9.75	60.0 ± 2.42	90	− 17.2 (− 21.95 to − 12.45)	< .001
Female	69.7 ± 8.73	57.0 ± 2.40	92	− 12.7 (− 17.40 to − 8.00)	< .001
30–39	75.9 ± 3.90	66.4 ± 4.03	32	− 9.5 (− 17.40 to − 1.60)	< .001
Male	78.6 ± 9.59	64.1 ± 5.39	20	− 14.5 (− 25.06 to − 3.94)	< .001
Female	71.2 ± 9.20	70.2 ± 6.03	12	− 1.1 (− 12.83 to 10.83)	0.689
40–49	75.1 ± 3.31	63.4 ± 3.84	41	− 11.7 (− 19.23 to − 4.17)	< .001
Male	77.5 ± 9.77	59.2 ± 5.39	24	− 18.3 (− 28.86 to − 7.74)	< .001
Female	70.9 ± 8.20	68.7 ± 5.09	17	− 2.2 (− 12.18 to 7.78)	0.258
50–59	73.7 ± 4.39	68.8 ± 4.35	30	− 4.8 (− 13.42 to 3.62)	0.348
Male	76.5 ± 9.56	75.2 ± 6.09	14	− 1.3 (− 13.24 to 10.64)	0.465
Female	68.6 ± 9.30	63.3 ± 5.9	16	− 5.3 (− 16.84 to 6.26)	0.023
60–69	68.5 ± 3.49	57.0 ± 3.2	36	− 11.5 (− 17.77 to − 5.23)	< .001
Male	74.2 ± 9.71	54.6 ± 4.41	17	− 19.6 (− 28.25 to − 10.95)	< .001
Female	64.0 ± 8.50	49.7 ± 4.66	19	− 14.3 (− 23.43 to − 5.17)	< .001
70+	60.4 ± 3.53	46.7 ± 2.42	43	− 13.7 (− 18.45 to − 8.95)	< .001
Male	65.0 ± 10.1	49.9 ± 3.25	16	− 15.1 (− 21.47 to − 8.73)	< .001
Female	55.5 ± 8.3	44.9 ± 3.33	27	− 10.6 (− 17.13 to − 4.07)	< .001

*n* number of individuals

The mean SMoS for non-operative patients was 62.1 (SD = 22.97) and 50.2 (SD = 21.25) for operative patients. Operative patients had a mean SMoS that was 11.9 points lower than non-operative patients (*p* < 0.0033, 95% CI − 19.88 to − 4.018).

Pearson’s correlation coefficient between SMoS and ODI, VAS and NDI were − 0.570 (*p* < .001, r^2^ = 0.3252), − 0.561 (*p* < .001, r^2^ = 0.314) and − 0.037 (*p* = 0.855, r^2^ = 0.001), respectively. This indicates a moderate negative correlation with ODI and VAS, but no correlation with NDI.

## Discussion

New patients presenting to a spinal surgery clinic displayed statistically significantly lower SMoS than expected from large population data samples and remained true when subjects were age and gender-matched to population data. Subgroup analysis revealed that patients who progressed to surgical intervention in the following 3 years had significantly worse SMoS than non-operative patients. This implies that the SMoS has differentiating power between patients with advancing disease severity, from none, to mild (not requiring intervention) to severe (requiring intervention) within the age and gender strata. Given the ambiguity of when to surgically intervene in conditions such as spinal stenosis, the SMoS could become a useful and quick tool in the future which could provide additional information to aid this decision.

Patients with high SMoS also had much lower physical disability according to well-established disability scores such as ODI and VAS, while low SMoS scores predicted high disability on the subjective measures of ODI and VAS. This is suggestive of the validity of the SMoS as a marker of physical disability and is in accordance with existing literature which suggests both walking speed and daily activity levels are reduced in the presence of diseases that affect the neurological and musculoskeletal systems [1, 6, 21]. While the SMoS should not replace these measures of disability, it can act as a useful adjunct to more holistically evaluate these patients.

Musculoskeletal disorders outside of the spine involved in walking, such as knee and hip osteoarthritis, also result in poor kinematic parameters including reduction in walking speed [22, 23]. Given the known association of walking quality with functional disability, and the additional association of walking quality with disease-specific disability scores in the present study, the SMoS may also be used to guide functional intervention by occupational and physiotherapy. The SMoS may also be used in the long-term monitoring of patient functional and disease status with a lower threshold for intervention. Given its ease of use and almost universal availability, there are very few barriers to the implementation of the SMoS in spinal surgical practice. Future studies in the fields of geriatrics, orthopaedics, mental health and other non-surgical neurological disorders will enhance the uptake of the SMoS as a routine practice as a clinical screening tool for both individual and population-based assessment.

### Inconsistencies

Notably, patients between 50 and 60 did not demonstrate a significant reduction in their SMoS compared to the expected values. Regarding female patients, recent literature has shown that the age of onset of mechanical low back pain is later for females than their male counterparts, corroborating findings that mechanical injuries in the spine appear decades earlier in men than women [24]. However, women are overrepresented in lower back pain statistics because of a combination of various lifelong events, such as childbearing, predisposition to mechanical instability at the L4/5 disk and hormonal changes that occur in the mid-50s that may lead to symptomatic deterioration of the spine. Additionally, male patients in the 50–59 age bracket demonstrated significantly higher SMoS than their trend would suggest. There is no evidence suggesting that males experience a sudden improvement in walking in this decade, and this is likely an anomalous result due to comparatively low sample size in this age bracket, a limitation of the present study that can be addressed in future studies. Moreover, NDI did not significantly correlate with SMoS despite other disability scores having good correlations. This is likely due to the anatomical implications of a cervical pathology being more likely to result in arm pain or weakness, while lower back issues tend to cause gait changes.

### Limitations

A limitation of this study is the use of population-based normative data as open-source, provided by the citizen science application *Argus* for age and gender-stratified DSC information [20]. Hence, our estimation of expected values of DSC is biased towards mid-high-income countries with access to smart devices. Additionally, this study assumed homogeneity amongst the various countries participating in the Althoff study, an assumption that was proven incorrect by the study in question. However, most data samples originated from the USA and Europe, where the average DSC was consistently 5000–6000 steps/day, giving a reasonable estimate of the expected population values for the patients in this study. Given the results of the Althoff study, a reasonable future direction for the SMoS would be similar trials to this, conducted in regions with a lower (Africa, Middle East, Southeast Asia) or higher (Russia, China and Scandinavian countries) DSC. Additionally, measurement of step count and walking speed by any device is contingent on the device and programme used to obtain the data. Furthermore, walking speed was measured from a walking bout in the vicinity of our spinal neurosurgery clinic, where the Hawthorne effect may confound results [3]. Although we were unable to account for this, future developments in smartphones may routinely measure walking speed using in-built health applications, allowing for future studies to investigate the SMoS in the patient’s daily life away from the Hawthorne effect. In addition, each patient only underwent one walking bout, affecting the reliability of our results. Future studies should investigate the SMoS using multiple walking bouts per patient. In addition, the accuracy of DSC may differ between smartphone types, preventing accurate comparisons of DSC between patients who use different smartphones. Nonetheless, differences of even 500 steps per day are unlikely to represent significant differences in daily physical activity (approximately equal to a 5-min difference in physical activity) [9]. While this is consistent with the SMoS as a general marker of physical health, future studies using other smart devices or fitness watches could investigate the SMoS using more accurate measurements of DSC. Finally, the cut-off values of 1.35 m/s (for walking speed) and 10,000 steps/day were based on the available literature [2, 9], which has been largely centred around population groups exhibiting contemporary living standards such as the USA. While this is likely applicable to our Australian study population, future studies can investigate the SMoS in more diverse population groups and cultures.

Commercial smart devices tend to have low levels of inaccuracy (3–10%) in step detection [25], but this increases to 40% in distance-based calculations using the GPS software [26] which the majority of smartphone-based WS calculations use. This is not the case for other smart devices or fitness watches. Thus, incorporation of smartphone-based health data using the SMoS or otherwise requires a more accurate assessment of WS and distance-based gait metrics. One potential using the SMoS is an application which measures step-count as per normal but uses a smart device’s in-built multiaxial accelerometer to calculate WS. This could be always running without significant battery dedication, or could operate on a reasonable threshold, e.g., only measuring once more than 10 consecutive steps have occurred and 10 s of activity has elapsed. This would capture longer walking bouts, giving an idea of a patient’s true functional WS with less battery consumption. It is likely that several short episodes of data capture to calculate walking speed will be adequate to provide a mean figure, rather than attempting to capture the speed of every step we take as this would be draining on processor and battery resources, and unnecessary. Notably, the Apple™ smartphones appear to have already implemented some accelerometer-based measurements, as they display gait metrics such as asymmetry and stance phases in the in-built health application.

## Conclusion

The detection and quantification of decline and recovery in physical and mental health status, across a broad range of pathologies, remains a challenge using a simple and single health measure, or score. The SMoS promises to be a tool that relays significant information about the individual that is easy for any health practitioner to understand and will assist with a range of healthcare decisions about a patient, while being easy to collect using readily available devices. Overall, there appears to be no major drawback to implementing the SMoS as a routine aspect of clinical evaluation, especially in gait disorders where it can be used to monitor disease progression and prognosticate.

## Supplementary Information

**Additional file 1.**

## Data Availability

The dataset supporting the conclusions of this article is included within the article and its additional files.

## Abbreviations

WS: Walking speed
DSC: Daily step count
ODI: Oswestry Disability Index
NDI: Neck Disability Index
VAS: Visual Analogue Score
SMoS: Simplified Mobility Score
